# Insulin glargine and the risk of cancer

**Published:** 2010-02

**Authors:** FA Ahmed

**Affiliations:** Isipingo Hospital, Natal, reports on data presented at the 2009 EASD congress

## Introduction

The concerns about a possible link between the use of Lantus (glargine) insulin and an increased risk of cancer were raised in the German study^1^ of around 127 000 insulin-treated patients in an insurance database. The research identified a statistically significant link between patients who had used Lantus insulin and those who had been diagnosed with cancer.

Compared with people using similar doses of human insulin, out of every 100 people who used Lantus insulin over an average of about one-and-a-half years,one additional person was diagnosed with cancer. Of particular note in this study was the finding that the increased risk of cancer was dose dependent. Therefore for patients given a dose of 10 units, Lantus insulin alone increased the risk of cancer by 9% compared with human insulin, but for a dose of 50 units, the increased risk was 31%.

Studies were then carried out using databases from Sweden, Scotland and the UK. The Swedish study^2^ found that compared with patients on insulins other than Lantus insulin, patients on Lantus alone had double the risk of breast cancer. The Scottish study found a non-significant increased risk for specifically breast cancers.^3^ The UK study found no link between insulin glargine and cancer.^4^

The researchers involved in all four studies agreed that these finding were not conclusive since the studies were observational and not clinical trials. The possibility that differences between groups of people were responsible for the different rates of cancer cannot be excluded. Further studies are needed before a final conclusion can be reached.

Jay Skyler, MD of the University of Miami, respresenting Sanofi-Aventis, reviewed the four articles appearing in the June 2000 Diabetologia.^1^–^4^ His conclusion was that one of the studies supported a special risk for insulin glargine above that of other insulin drugs.^2^ Skyler also said that cancer incidences among patients treated with insulin glargine in uncontrolled analyses did not differ from those reported in the CDC’s Surveillance, Edpiemiolgy and End Results database after adjusting for patient age.

Novo Nordisk proposed David Russel-Jones, MD of the University of Surrey in England, who reviewed clinical trial data on insulin detemir compared with two other insulin-based agents, including glargine, covering some 9 000 patients. He said that cancer incidence among those receiving detemir was significantly lower among patients treated with NPHinsulin. There was no significant difference in cancer rates between the detemir and glargine analogues.

At a well-attended symposium at the EASD, researchers reviewed the existing data on cancer and diabetes drugs, including but not limited to insulin and its analogues. What had emerged most clearly, the researchers agreed, was that patients receiving insulin-based agents, and to a lesser extent sulfonylurea drugs, appeared more likely to be diagnosed with cancer.

Jeffrey Johnson, of the University of Alberta in Edmonton, Canada, reviewed data he and other scientists had reported on earlier that year, covering patients in Canada’s Saskatchewan province from 1995 to 2006. Compared with patients receiving metformin monotherapy, those taking sulfonylurea drugs were at a 30% greater risk of being diagnosed with cancer, Johnson said.

The data showed that patients receiving insulin with fewer than 12 prescriptions per year had a significant 67% increase in the likelihood of cancer diagnosis, relative to those on sulfonylurea monotherapy. Those receiving more insulin prescriptions had a whopping sevenfold increase in risk.

The researchers generally agreed on several other points:

The retrospective data now available do not permit conclusions about causality.
The current evidence suggesting insulin glargine-treated patients are at more risk for cancer than patients receiving other insulin-based agents is weak.
No association has been seen between metformin or thiazolidinediones and increased cancer risks.
Some studies have suggested that metformin can reduce cancer risk, both axalone and in combination with insulin glargine.
It is possible that insulins may promote cancer through their action as growth factors.
It is also possible, however, that patients treated with insulin are at risk for cancer because of the underlying disease, not because of the drugs.
No associations were shown in younger type 1 diabetics who most benefit from insulin analogue therapy.

## Conclusion

Prof UH Smith, president of the EASD5 said that the associations between insulin products and cancer risk fell far short of demonstrating causality. They deserve more research but do not warrant any changes on treatment protocols at this time, he said.

The EASD does not recommend that patients should stop taking insulin glargine on the basis of evidence presented in these studies. Patients do however have the option of using long-lasting human insulin or a mixture of short- and long-acting human insulin twice a day instead of the once-daily analogue. Especially, patients who already have cancer or women with a family history of breast cancer may wish to consider this option.
